# Adverse events during radical prostatectomy and their association with recurrence and death

**DOI:** 10.1007/s00345-025-05932-7

**Published:** 2025-09-23

**Authors:** Sofia Erestam, Ying Li, Eva Angenete, Anders Bjartell, Carolina Ehrencrona, Jonas Hugosson, Anna Lantz, Peter Wiklund, Eva Haglind

**Affiliations:** 1 Institute of Health and Care science, University of Gothenburg, Gothenburg, Sweden; 2 Department of Surgery, SSORG-Scandinavian Surgical Outcomes Research Group, Institute of Clinical Sciences, Sahlgrenska Academy, University of Gothenburg, Gothenburg, Sweden; 3 Biostatistics, School of Public Health and Community Medicine, Institute of Medicine, Sahlgrenska Academy, University of Gothenburg, Gothenburg, Sweden; 4 Region Västra Götaland, Department of Surgery, SSORG Sahlgrenska University Hospital/Östra, 416 85 Gothenburg, Sweden; 5 Department of Urology, Skåne University Hospital, Malmö, Sweden; 6 Department of Translational Medicine, Faculty of Medicine, Lund University, Malmö, Sweden; 7 Department of Urology, Institution of Clinical Sciences, Sahlgrenska Academy, University of Gothenburg, Gothenburg, Sweden; 8 Department of Urology, Sahlgrenska University Hospital, Gothenburg, Sweden; 9 Department of Molecular Medicine and Surgery, Karolinska Institutet, Stockholm, Sweden; 10 Department of Medical Epidemiology and Biostatistics, Karolinska Institutet, Stockholm, Sweden; 11 Department of Urology, Icahn School of Medicine, Mount Sinai Health System, New York City, NY US

**Keywords:** Adverse events, Prostate cancer, Surgical technique, Radical prostatectomy, Risk factors, Oncological outcomes

## Abstract

**Objectives:**

The objective was to explore if adverse events during radical prostatectomy for prostate cancer were associated with oncological outcomes. A further objective was to identify risk factors for adverse events.

**Methods:**

A post-hoc study nested in a prospective, controlled trial of radical prostatectomy by robot assisted laparoscopic or open retropubic approach in Sweden. Adverse events during surgery were collected from clinical record forms (CRFs) filled out by the surgeon at operation. Recurrence was identified from CRFs and patient reports, cause of death through the Swedish National Cause of Death Register. Recurrence was defined as undetectable PSA 6–12 weeks after prostatectomy followed by PSA > 0.25 ng/ml or treatment for prostate cancer recurrence. Cox regression was used to explore associations between exposure and outcome.

**Results:**

One/more adverse events occurred during 39% (1356/ 3444) of operations. Adverse events were associated with recurrence, but not with all-cause or prostate cancer specific mortality. Intraoperative extensive bleeding and difficulties during dissection were associated with recurrence. Risk factors included age at surgery, history of TUR-P or abdominal surgery, teaching, prostate weight and lymph node dissection. Limitations included the low number of observations (deaths), particularly in subgroup analyses and hospital volume. This study should be regarded as explorative.

**Summary:**

In this explorative study adverse events during radical prostatectomy were associated with increased risk for recurrence.

**Supplementary Information:**

The online version contains supplementary material available at 10.1007/s00345-025-05932-7.

## Introduction

Prostate cancer is the most common type of cancer in Sweden [1]. Treatment aimed for cure could be by radiotherapy or surgery, where radical prostatectomy as treatment of localized prostate cancer was found to decrease prostate cancer specific mortality compared with watchful waiting in an early trial but more recently a comparison between active surveillance, surgery or radiotherapy found no significant diffences [2–4]. Initially radical prostatectomy used the open approach, but over the last 20 years this has almost been replaced by the less invasive approach robot assisted laparoscopy.

LAPPRO trial is a prospective, controlled, multicenter, non-randomized trial with the aim to compare two surgical techniques: open radical prostatectomy (ORP) and robot assisted laparoscopic prostatectomy (RALP) [5]. Previous studies in the LAPPRO trial showed no difference between the two surgical techniques regarding primary outcome urinary incontinence at 12 or 24 months, but that RALP resulted in a small benefit in erectile function [6, 7]. After 12 years follow-up prostate cancer-specific mortality was lower among patients operated by RALP technique (2.0%) compared with open surgery (4.5%) [8].

Adverse events during surgical procedures are not always systematically documented in studies on surgical techniques. A meta-analysis comparing different surgical techniques for radical prostatectomy concluded that adverse events were less common using the RALP technique [9]. Recurrence of cancer is a burden for the patient, both psychologically and as a result of side effects from treatment. Known risk factors for recurrence after radical prostatectomy are higher age, tumor grade by Gleason classification, advanced tumor stage, and positive surgical margin, whereas nerve sparing technique was not associated with recurrence [10–12].

The hypothesis is that difficulties during surgery could reflect on the radicality (“R0”) of the procedure and/or the amount of traumatized tissue in the operating area, which could affect the imunological reactions to circulating cancer cells.

The aim of this explorative study was to investigate associations between adverse events during surgery and oncological outcomes in terms of recurrence, prostate cancer specific and all-cause mortality within 12 years, for the entire cohort and for RALP and ORP separately. An additional aim was to identify preoperative risk factors associated with adverse events.

## Methods

This study is a post-hoc analysis nested within the LAPRO trial. LAPPRO was a prospective, controlled, multicenter trial with the primary aim to compare urinary incontinence between open radical prostatectomy and robot assisted laparoscopic prostatectomy after 12 months [5]. All patients at the participating 14 centers scheduled for prostatectomy due to localized prostate cancer were asked about participation, and all who consented were included. After informed consent patients were included between 1st of September 2008 and 7th of November 2011. Inclusion criteria at baseline were as follows: age < 75 years, prostate specific antigen < 20 ng/ml, clinical tumor stage < T4, no diagnosis of metastatic disease. Exclusion criteria were applied at analyses. Seven centers used ORP and seven centers RALP as surgical technique. The trial was non-randomized.

### Data collection

After inclusion patients were followed for 12 years, using seven clinical record forms answered by health care personnel and seven questionnaires distributed to patients as described earlier [5]. The intraoperative clinical record form was filled out by the operating surgeon immediately after surgery and included detailed questions on every step of the procedure, with specific questions about perceived difficulties and adverse events. Cause of death and date of death were retrieved (August 10, 2023) from the National Cause of Death Register (Swedish Board of Health and Welfare). Data on recurrence was collected by CRFs at follow up 12 or 24 months after surgery or by patients as reported in the questionnaires.

### Objectives

The primary objective for this exploratory study was the comparison of recurrence at 12 months post-operatively until the 12-year follow-up for two groups of patients: one group where adverse events occurred during surgery, and one group where no adverse events were recorded during the operation. This analysis was made for the entire cohort and for RALP and ORP separately.

The secondary objective was to investigate if adverse events during surgery were associated with all-cause mortality or prostate cancer specific mortality within 12 years from surgery, for the entire cohort.

Further objectives were to identify preoperative risk factors associated with adverse events for the entire cohort.

### Outcomes

The primary endpoint recurrence was defined as an initially undetectable PSA value (< 0.25 ng/ml) at 6–12 weeks after surgery followed by measurable PSA level at any time during follow-up or by treatment due to recurrence at any time during follow-up (Supplement Table 1) [13]. Secondary outcomes were all-cause mortality and prostate cancer specific mortality.

### Adverse events

Intraoperative adverse events have previously been defined as “any significant deviation from the standard surgical procedure, which was unforeseen and/or unintentional, and which significantly complicated the surgery, prolonged the duration of the surgery and/or negatively affected the way the surgery was executed” [14].

Adverse events were collected from the intraoperative CRF (Supplementary material), which was filled out by the surgeon immediately after surgery, where objective measures such as operating time or perioperative bleeding as well as subjective variables such as surgeon experiences (“difficulties during dissection”) were included. The CRF was content, and face validated by experts before being used in LAPPRO. Experiences during the pilot study were also used for revision.

Adverse events were grouped into the following seven categories:

1. Prolonged operating time was defined as the longest 10% of the operating duration in ORP and RALP groups respectively. A patient was identified as having an adverse event associated with extended operating time if the operation time exceeded 171.2 min in ORP or 264 min in RALP.
2. Extensive perioperative bleeding was defined as the highest 10% of bleeding volume in ORP and RALP groups respectively. A patient was identified as having an adverse event related to extensive bleeding if the blood loss exceeded 1300 ml in ORP or 400 ml in RALP.
3. Cutting anastomotic suture/s (yes/no).
4. Difficulties when dissecting (yes/no).
5. Any defined problem during the procedure (yes/no).
6. Adhesiolysis in the abdomen (yes/no).
7. Others, any of the following: conversion to open procedure, repair of damage to other organ, additional surgical procedure (yes/no).

Exposure to adverse events was measured in two ways:

- (A) A combined measure: any of the adverse events as listed above, dichotomous (Yes / No).
- (B) Each of the adverse events separately as an independent exposure.

### Statistical methods

The associations between adverse events during surgery and outcomes, including recurrence, all-cause mortality and prostate cancer specific mortality were investigated with Cox regressions. Follow-up for the time-to-event analyses (for mortality and recurrence) began at the time of surgery. Recurrence was considered as time to event outcome, with follow-up at 12 and 24 months, and 6, 8 and 12 years. The exact date of the events (PSA increase or initiation of adjuvant/salvage treatment) or censoring (dropout, mortality or no event at 12 years) are interval-censored between the current and previous follow-up dates. For this reason, the event time was set to the midpoint between the two consecutive follow-up dates. For example, if an elevated PSA was reported at 24 months but not at 12 months, it was assumed to have occurred at 18 months. For mortality, patients were followed until at their date of death as per the Swedish Cause of Death Register. Patients who did not die were censored at the data retrieval date. The analysis was adjusted for the clinical T stage (T1-T3), preoperative PSA, Gleason score on biopsy, length of cancer in preoperative biopsy cores, and prostate weight. The proportion hazard assumption was assessed using the score test (cox.zph() function in R). Since there was an indication of a violation of the proportional hazards for preoperative PSA, this variable was included in time-dependent format using the tt() function in coxph package in R. This investigation was conducted across the entire study cohort and within the two sub-groups, robot assisted laparoscopic and open surgery, respectively.

In view of the exploratory nature of the study, no correction for multiple testing was performed.

Backward variable selection utilizing Akaike information criterion (AIC) was employed to identify the predictive variables for the combined adverse event during surgery. The combined adverse event, as defined earlier, encompassed any adverse event from the seven groups. The variables were: age at surgery (age < 60, 60-<70, >=70), BMI (> 30</=30), TUR-P in history (Yes/No), abdominal surgery in history (Yes/No), clinical T-stage (T1-T3), preoperative Gleason score ( < = 7, >=8), surgeon experience (number of surgeries performed), surgeon participation in teaching during surgery, prostate weight (g) (0- <20; 20- <40; 40- <60; 60- <80; >/=80) and lymph node dissection (Yes/No). Bootstrap was used to perform stability investigations. The basic idea of bootstrap stability investigation is to draw N resamples from the original data set and to repeat variables selection in each of the resamples. In this study, we drew 100 sets of resamples. Variable of importance (VIF) was presented, which indicated how often a certain variable was selected in the model in the 100 simulations.

All the analyses were conducted in R version 4.3.2.

### Ethics

The Regional Ethics Review Board in Gothenburg, Sweden, approved the study 2007 (No 277-07) with additional permissions 2014-07-28 plus 2015-04-24 for continued follow-up and data retrieval. The trial was conducted in accordance with the Declaration of Helsinki. After recieving information about the trial and having been given the opportunity to ask questions and receive answers from local investigators, all participants gave signed consent. The trial was registered at ISRCTN 07/02/2008 (ISRCTN06393679).

## Results

**Fig. 1:**
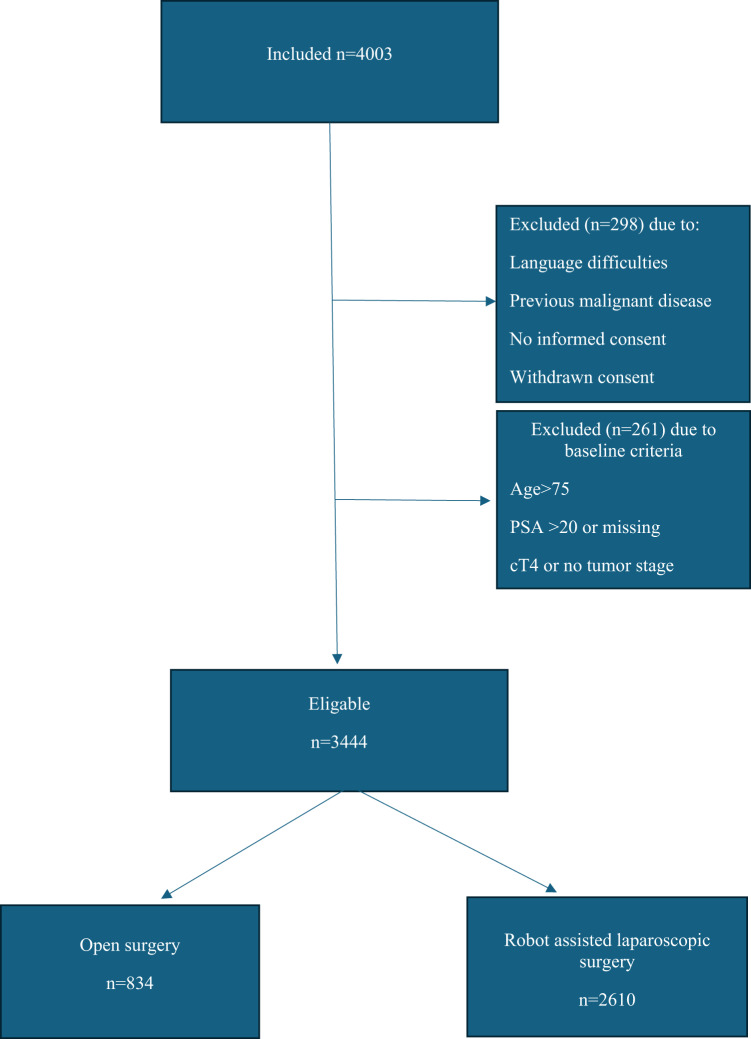
Study flow chart

LAPPRO included 4003 patients who underwent radical prostatectomy for localized prostate cancer out of which 834 were eligible for analyses after ORP and 2610 after RALP (Fig. 1).

Missing data in an independent exposure variable included in the combined analysis of adverse events (any adverse event) lead to exclusion from the analysis. Details about missing data is given in Supplementary Table 2.

We found that in 1356 out of 3444 (39.4%) procedures, surgeons reported one or more adverse events. Patients with intraoperative adverse events were somewhat younger than patients with no such adverse events but had similar BMI (Table 1). The group not possible to include in analyses (“Missing”) did not differ from those with or without reported adverse events on age, BMI or tumor factors but had shorter operating time and more experienced surgeons (Table 1). Operating time was longer in RALP whereas perioperative bleeding was higher in the ORP group (Table 1).

**Table 1:** Patient characteristics for groups with and without adverse events, as well as the group of patients with one or more adverse event variables missing, regardless of type of surgery and separately for patients operated by open retropubic prostatectomy (ORP) and robot assisted laparoscopic prostatectomy (RALP), during radical prostatectomy

Characteristic	No adverse event *n* = 935	Adverse Event *n* = 1356	Missing *n* = 1153	ORP, *n* = 834	RALP, *n* = 2610
Age at surgery, years^a^	62 (57, 66)	64 (59, 67)	63 (59, 67)	63 (59, 67)	63 (58, 67)
Preoperative BMI, kg/m^2^, ^a^	25 (24, 28)	26 (25, 29)	26 (24, 28)	26 (25, 28)	26 (24, 29)
Missing (n)	89	175	170	86	348
Clinical_T_stage, number (%)					
T1	602 (64%)	811 (60%)	688 (60%)	555 (67%)	1,546 (59%)
T2	313 (33%)	503 (37%)	423 (37%)	250 (30%)	989 (38%)
T3	20 (2.1%)	42 (3.1%)	42 (3.6%)	29 (3.5%)	75 (2.9%)
Preoperative Gleason score > = 8, number (%)	33 (3.5%)	90 (6.7%)	70 (6.1%)	47 (5.6%)	146 (5.6%)
Missing (n)	5	3	6	1	13
Preoperative PSA, ng/ml^a^	5.7 (4.2, 8.1)	6.4 (4.6, 9.3)	6.0 (4.5, 8.6)	6.3 (4.5, 9.1)	6.0 (4.4, 8.7)
Length of cancer in biopsy, mm^a^	7 (4, 14)	8 (4, 16)	8 (4, 17)	7 (3, 15)	8 (4, 16)
Missing (n)	44	94	92	84	146
Prostate weight, g^a^	40 (33, 50)	44 (36, 56)	43 (35, 52)	44 (36, 54)	42 (34, 53)
Missing (n)	5	15	26	14	32
Operation time, min^a^	165 (141, 193)	196 (154, 247)	115 (78, 158)	94 (75, 135)	181 (151, 219)
Missing (n)	0	180	320	39	461
Lymph node dissection performed, number (%)	22 (2.4%)	116 (8.6%)	67 (5.8%)	92 (11%)	113 (4.3%)
Intraoperative bleeding, ml^a^	100 (50, 200)	200 (100, 500)	250 (100, 550)	550 (350, 800)	100 (50, 200)
Missing (n)	0	49	153	17	185
Surgeon experience, number^a^	181 (103, 290)	152 (71, 310)	241 (110, 818)	511 (126, 1,153)	162 (81, 283)
Missing (n)	7	9	12	15	13
Recurrence, n	178	350	260	194	594
All-cause mortality, n	91	226	172	135	354
Prostate cancer specific mortality, n	10	38	42	38	52

^a^Median (IQR)

For the outcome recurrence patient reports in questionnaires were used at long-term follow up. Answering rate was 67% for the latest questionnaire used in the combined outcome “recurrence”. Mortality data has virtually no missing values, due to the nature of the Cause of Death Register.

Any adverse event during surgery (combined level) was associated with increased risk for recurrence within 12 years of surgery in the entire group of patients (adjusted HR 1.38; CI 1.15–1.66; p 0.001) (Table 2), and after RALP (Table 2) but not after ORP (Table 2). When analyzing the seven types of adverse events separately in the entire cohort, there were associations between three of them and recurrence: perioperative extensive bleeding (adjusted HR 1.64; CI 1.29–2.10; *p* < 0.001), difficulties when dissecting (adjusted HR 1.39; CI 1.13–1.71; *p* = 0.002) (Table 2). In the ORP subgroup extensive perioperative bleeding was associated with recurrence and in the RALP subgroup this was also the case, as was “difficult dissection” (Table 2). In a supporting analysis total blood loss volume was associated with recurrence in those operated by open technique (adjusted HR 1.0006 (1.0005,1.001), *p* < 0.0001) and in the RALP group (adjusted HR 1.001 (1.0004,1.001), *p* < 0.0001).

**Table 2:** Association between adverse events during surgery and recurrence within 12 years follow up comparing groups with or without adverse events

	Hazard ratio	95% CI^a^		*p*-value
Any type of surgery				
Combined adverse events group 1–7	1.38	1.15	1.66	0.001
Combined adverse events group 2–7	1.39	1.17	1.65	< 0.001
1 Prolonged operating time (Yes/ No)	1.11	0.87	1.41	0.402
2 Extensive perioperative bleeding (Yes/No)	1.64	1.29	2.10	< 0.001
3 Cutting anastomotic suture/s (Yes/No )	1.02	0.80	1.32	0.854
4 Difficult dissection (Yes/No)	1.39	1.13	1.71	0.002
5 Any defined problem (Yes/No)	1.14	0.96	1.35	0.144
6 Adhesiolysis in the abdomen (Yes/No)	1.11	0.65	1.89	0.702
7 Others, any of the following: conversion to open procedure, repair of damage to other organ, additional surgical procedure (yes/no)	0.80	0.33	1.93	0.616
Open radical prostatectomy (ORP)				
Combined adverse events group 1–7^b^	-	-	-	-
Combined adverse events group 2–7	2.88	0.90	9.28	0.075
1 Prolonged operating time (Yes/ No)	1.14	0.73	1.79	0.556
2 Extensive perioperative bleeding (Yes/No)	1.97	1.30	2.98	0.001
3 Cutting anastomotic suture/s (Yes/No )	1.25	0.72	2.16	0.433
4 Difficult dissection (Yes/No)	1.62	0.51	5.15	0.414
5 Any defined problem (Yes/No)	1.30	0.87	1.93	0.198
6 Adhesiolysis in the abdomen^b^ (Yes/No)	-	-	-	-
7 Others, any of the following: conversion to open procedure, repair of damage to other organ, additional surgical procedure (yes/no)	1.40	0.44	4.38	0.568
Robot assisted laparoscopic radical prostatectomy (RALP)				
Combined adverse events group 1–7	1.35	1.11	1.64	0.002
Combined adverse events group 2–7	1.33	1.11	1.60	0.002
1 Prolonged operating time (Yes/ No)	1.10	0.82	1.46	0.524
2 Extensive perioperative bleeding (Yes/No)	1.50	1.10	2.03	0.009
3 Cutting anastomotic suture/s (Yes/No )	0.99	0.74	1.31	0.924
4 Difficult dissection (Yes/No)	1.41	1.14	1.75	0.002
5 Any defined problem (Yes/No)	1.11	0.92	1.35	0.266
6 Adhesiolysis in the abdomen (Yes/No)	1.21	0.71	2.05	0.493
7 Others, any of the following: conversion to open procedure, repair of damage to other organ, additional surgical procedure (yes/no)	0.47	0.12	1.87	0.282

Adjusted for clinical T stage, preoperative PSA, Gleason score on biopsy and prostate weight

^a^Indicates confidence interval

^b^Not analyzed due to missing values

There was an association between adverse events during radical prostatectomy and all-cause mortality at 12 years follow up (adjusted HR 1.57; CI 1.22–2.01; *p* < 0.001) analyzing the entire cohort and the combination of all seven types of adverse events (Table 3). Difficulties at dissection were associated with all-cause mortality (adjusted HR 1.34(CI1.03-1.75; *p* < 0.029). Adverse events were not associated with prostate cancer specific mortality (Supplement Table 3).

**Table 3:** Association between adverse events during radical prostatectomy and all-cause mortality after 12 years follow up comparing groups with or without adverse events

	Hazard ratio	95% CI^a^		*p*- value
Combined adverse events group 1–7	1.57	1.22	2.01	< 0.001
Combined adverse events group 2–7	1.52	1.21	1.92	< 0.001
1 Prolonged operating time (Yes/ No)	1.17	0.88	1.56	0.279
2 Extensive perioperative bleeding (Yes/No)	1.34	0.98	1.84	0.065
3 Cutting anastomotic suture/s: (Yes/No )	1.00	0.73	1.37	0.979
4 Difficult dissection (Yes/No)	1.34	1.03	1.75	0.029
5 Any defined problem (Yes/No)	1.19	0.96	1.46	0.109
6 Adhesiolysis in the abdomen (Yes/No)	1.13	0.58	2.19	0.714
7 Others, any of the following: conversion to open procedure, repair of damage to other organ, additional surgical procedure (yes/no)	1.14	0.42	3.04	0.801

Adjusted for clinical T stage, preoperative PSA, Gleason score on biopsy and prostate weight

^a^Indicates confidence interval

We identified pre- or perioperative risk factors for adverse events during surgery (Table 4), where age at surgery (OR 1.34; CI 1.00–1.55), BMI (OR 1.95; CI 1.43–2.71), prostate weight (OR 1.38; CI 1.22–1.54), TUR-P (OR 3.7; CI 1.82–9.53), prior abdominal surgery (OR 1.31; CI 1.00–1.75), teaching during surgery (OR 1.3; CI 1.00–1.58), and lymph node dissection (OR 4.72; CI 2.75–8.61) were variables of importance (VIF). When removing operating time from the equation teaching disappeared as a variable of importance (Table 4).

**Table 4:** Risk factors for adverse events, combined group 1–7 and combined group 2–7 comparing groups with and without adverse events

	combined 1–7			combined 2–7		
Risk factor	VIF^a^	OR^b^	95% CI^c^	VIF^a^	OR^b^	95% CI^c^
Age	96	1.34	1, 1.55	98	1.28	1.13, 1.5
BMI	100	1.95	1.43, 2.71	99	1.98	1.55, 2.72
Previous TUR-P	100	3.7	1.82, 9.53	98	2.99	1.56, 5.89
Previous abdominal surgery	75	1.31	1, 1.75	80	1.32	1, 1.69
cT	6	1	0.84, 1	18	1	0.87, 1.21
Preop_Gleason_score	23	1	1, 1.8	15	1	0.63, 1.43
Prostate_weight	100	1.38	1.22, 1.54	100	1.35	1.24, 1.51
Lymph_node dissection	100	4.72	2.75, 8.61	98	2.3	1.52, 3.83
Surgeon experience	19	1	1, 1	16	1	1, 1
Teaching in OR	92	1.3	1, 1.58	16	1	0.9, 1.21

Adjusted for clinical T stage, preoperative PSA, Gleason score on biopsy and prostate weight

^a^VIF: variable of importance. In 100 simulations, the variable was selected this number of times in the model

^b^Indicates Odds Ratio

^c^Indicates confidence interval

## Discussion

In this post-hoc exploratory analysis, nested in a prospective comparative trial, adverse events during radical prostatectomy for prostate cancer were associated with an increased risk for recurrence. Specifically, three of the defined adverse events, perioperative bleeding, difficulty at dissection and reporting “any defined problem during surgery” were separately related to increased risk for recurrent disease.

Adverse events after abdominal surgery have been associated with a higher risk of postoperative mortality, morbidity and increased postoperative length of stay [15]. An association between intraoperative adverse events and local recurrence within five years after surgery for rectal cancer has been reported [14].

The list of adverse events expressed as questions in the clinical record form (CRF) filled out by the surgeons at the time of the surgery included objective measures. Several questions had answer alternatives Yes or No, without follow up questions about extent. Other questions represented subjective experiences such as difficulty during dissection, any problem during the operation, all answered by Yes/No. As the answers to questions were categorical it was not possible to extend analyses to how much or how difficult. There is as always a need to balance the amount of details asked for and the expected compliance in filling out questionnaires.

Dissection difficulties were associated with recurrence in the entire cohort as well as in the subgroup operated by robot assisted laparoscopy. Part of the explanation for the somewhat diverse findings could be lack of power, as number of events decreased with subgrouping, especially in the openly operated group. Extensive blood loss during surgery was associated with recurrence for both robot assisted laparoscopic and open surgery, indicating that this association was not technique specific. It should be noted that we defined “blood loss” as the top 10% percentile of perioperative bleeding for each technique, and as blood loss was significantly smaller in RALP compared with ORP “extensive blood loss volume” was different in the two techniques. We suggest that blood loss defined as the top 10% represented technical difficulties during the surgical procedure rather than the actual volume of blood lost. “Dissecting difficulties” and “Any defined problem during surgery” were also associated with worse oncological outcome which would suggest that our interpretation of a mediation between excessive blood loss and oncological outcome could be correct, that the blood loss should be regarded as proxy for difficulties during surgery. Our findings indicate that meticulous surgery without adverse events is possibly of importance, also in long term outcomes. Although adverse events and risk factors were associated with higher risk of recurrence in our analyses, the analyses of mortality suffer from lack of power due to the restricted number of events (deaths) [8].

We have earlier described an inverse relationship between surgeon satisfaction measured directly after the operation and intraoperative events such as technical difficulties and intraoperative events as described by the surgeon at the time [16]. The present results add knowledge regarding the implication of intraoperative events for oncological outcomes. It could be suggested that surgeon satisfaction could be a relevant quality outcome of radical prostatectomy. Whether surgeon dissatisfaction could be used as “proxy” for a need for more frequent follow-up, needs further studies.

In the analyses of risk factors for adverse events during surgery several factors of importance were found such as a history of surgery on the prostate or in the abdomen, a large prostate as well as lymph node dissecion during the procedure. Further studies may lead to identification of a prediction model that could assist preoperative considerations of how to best prepare and perform the surgical procedure.

We have earlier reported a difference regarding perioperative blood loss volume between the two techniques, in favor of RALP [17], similar to most reports about laparoscopic surgery regardless of type of procedure [18, 19]. This phenomenon has been attributed in part to the necessity to perform “bloodless surgery” as bleeding during any type of laparoscopy results in decreased visibility. Another hypothesis is that the positive pressure inside the abdominal cavity during any laparoscopic procedure, by the insufflation of carbon dioxide at a positive pressure, will overcome the pressure in venules/small veins and thus result in a rapid cessation of small bleeding points.

A strength in this study was the intraoperative clinical record form filled out by the operating surgeon, in immediate connection with the operation. The recording of adverse events is both systematic and detailed, and in combination with immediate reporting could be taken as an indication of high validity of data, compared with retrospective study designs using medical records [14]. Further strengths were the cohort size and the multicenter design. The lack of established definitions of the various adverse events should be regarded as a limitation, to some extent balanced by distinct questions and answer alternatives in the clinical record form. The relatively high number of missing is a limitation in analyses of combined variables (“any adverse event”) as is the non-randomized design. Another limitation relates to a low number of events (deaths), particularly in subgroup analyses. A further limitation could be that neither surgeon experience nor center were included in the statistical models. This study is explorative in nature, and the outcomes were not the primary endpoint of the LAPPRO trial.

## Conclusion

This explorative study found associations between adverse events during radical prostatectomy and oncological outcome. Efforts are needed to establish a causal relationship.

### Take home message

Adverse events during radical prostatectomy were associated with increased recurrence and all-cause mortality at follow up at 12 years after surgery. It is possible that actions to counteract perioperative bleeding and/or dissection difficulties could be of value.

## Supplementary Information

Below is the link to the electronic supplementary material.

Supplementary Material 1

## Data Availability

No datasets were generated or analysed during the current study.
