# Supplementary material for: Adverse events during radical prostatectomy and their association with recurrence and death
Source: World J Urol. 2025 Sep 23;43(1):570. doi: 10.1007/s00345-025-05932-7 (PMC12457513; doi:10.1007/s00345-025-05932-7)
Supplement: Supplementary file 1 — Supplementary Material 1 [file 345_2025_5932_MOESM1_ESM.docx]

SUPPLEMENT

Supplement Table 1 Recurrence defined as one or more of the below listed criteria, after undetectable PSA at 6-12 weeks.

| Time point | PSA >0.25ng/ml | Radio-/Hormonal -/Chemo-/Other therapy for recurrence | | | |
| --- | --- | --- | --- | --- | --- |
| 12 months | yes | yes | Yes | yes | yes |
| 24 months | yes | yes | Yes | yes | yes |
| 6 years | yes | yes | Yes | yes | yes |
| 8 years | yes | yes | Yes | yes | yes |
| 12 years | yes | yes | Yes | yes | yes |

Supplement Table 2: Number of participants without/with adverse events during surgical procedure and missing

|  |  | not adverse (0) | adverse (1) | Missing |
| --- | --- | --- | --- | --- |
| 1 | **Operating time** | **2651** | **293** | **500** |
| 2 | **Perioperative bleeding** | **2993** | **249** | **202** |
| 3 | Did any of the sutures/takes in bladderneck cut | 3 297 | 76 | 71 |
|  | Did any of the sutures/takes in urethra cut | 3239 | 142 | 63 |
|  | Difficulties with the anastomosis | 3258 | 117 | 69 |
|  | **Combined group 3 (anastomosis)** | **3062** | **299** | **83** |
| 4 | Difficulties at dissection of the prostate | 3302 | 93 | 49 |
|  | Difficulties at dissection of the bladder | 2602 | 33 | 809 |
|  | Difficulties at dissection of bladderneck/prostate | 3219 | 173 | 52 |
|  | Difficulties at dissection of tertius | 3331 | 52 | 58 |
|  | Difficulties at dissection of neurovascular bundles | 2551 | 162 | 731 |
|  | Difficulties at dissection of vesicula seminales | 3181 | 162 | 101 |
|  | **Combined group 4 (difficulties at dissection)** | **1809** | **489** | **1146** |
| 5 | **Any of defined complications** | **2703** | **698** | 43 |
| 6 | **Adhesiolysis** | 3382 | **62** |  |
| 7 | Conversion to open procedure | 3441 | 3 |  |
|  | Repair damage to other organ | 3431 | 13 |  |
|  | Additional procedure | 3439 | 5 |  |
|  | **Combined group 7 (other)** | **3423** | **21** |  |

Supplement Table 3 Association between adverse events during radical prostatectomy and prostate cancer specific mortality at 12 years follow-up

|  | Hazard ratio | 95% CI^1^ | | p value |
| --- | --- | --- | --- | --- |
| Combined adverse events group 1-7 | 1.75 | 0.85 | 3.58 | 0.126 |
| Combined adverse events group 2-7 | 1.68 | 0.88 | 3.20 | 0.118 |
| 1 Prolonged operating time (Yes/ No) | 1.30 | 0.72 | 2.33 | 0.379 |
| 2 Extensive perioperative bleeding (Yes/No) | 1.43 | 0.65 | 3.12 | 0.377 |
| 3 Cutting anastomotic suture/s: (Yes/No ) | 0.99 | 0.46 | 2.16 | 0.982 |
| 4 Difficult dissection (Yes/No) | 2.49 | 1.29 | 4.80 | 0.007 |
| 5 Any defined problem (Yes/No) | 0.73 | 0.44 | 1.23 | 0.236 |
| 6 Adhesiolysis in the abdomen (Yes/No) | 2.32 | 0.56 | 9.54 | 0.243 |
| 7 Others, any of the following: conversion to open procedure, repair of damage to other organ, additional surgical procedure (yes/no) | ** | ** | ** | ** |

Adjusted for clinical T stage, preoperative PSA, Gleason score on biopsy and prostate weight

^1^Indicates confidence interval

** Cannot be estimated due to limited number of observations
